# The Single Item Literacy Screener: Evaluation of a brief instrument to identify limited reading ability

**DOI:** 10.1186/1471-2296-7-21

**Published:** 2006-03-24

**Authors:** Nancy S Morris, Charles D MacLean, Lisa D Chew, Benjamin Littenberg

**Affiliations:** 1 College of Nursing and Health Sciences, University of Vermont, Burlington, Vermont, USA; 2 College of Medicine, University of Vermont, Burlington, Vermont, USA; 3 Harborview Medical Center, University of Washington, Seattle, Washington, USA

## Abstract

**Background:**

Reading skills are important for accessing health information, using health care services, managing one's health and achieving desirable health outcomes. Our objective was to assess the diagnostic accuracy of the Single Item Literacy Screener (SILS) to identify limited reading ability, one component of health literacy, as measured by the S-TOFHLA.

**Methods:**

Cross-sectional interview with 999 adults with diabetes residing in Vermont and bordering states. Participants were randomly recruited from Primary Care practices in the Vermont Diabetes Information System June 2003 – December 2004. The main outcome was limited reading ability. The primary predictor was the SILS.

**Results:**

Of the 999 persons screened, 169 (17%) had limited reading ability. The sensitivity of the SILS in detecting limited reading ability was 54% [95% CI: 47%, 61%] and the specificity was 83% [95% CI: 81%, 86%] with an area under the Receiver Operating Characteristics Curve (ROC) of 0.73 [95% CI: 0.69, 0.78]. Seven hundred seventy (77%) screened negative on the SILS and 692 of these subjects had adequate reading skills (negative predictive value = 0.90 [95% CI: 0.88, 0.92]). Of the 229 who scored positive on the SILS, 92 had limited reading ability (positive predictive value = 0.4 [95% CI: 0.34, 0.47]).

**Conclusion:**

The SILS is a simple instrument designed to identify patients with limited reading ability who need help reading health-related materials. The SILS performs moderately well at ruling out limited reading ability in adults and allows providers to target additional assessment of health literacy skills to those most in need. Further study of the use of the SILS in clinical settings and with more diverse populations is warranted.

## Background

Optimal health care requires an informed and active patient who can seek, obtain, and understand health information. Health literacy, a concept that focuses specifically on literacy concerns within the context of health, has many components including numeracy, oral literacy, print literacy, and cultural and conceptual knowledge [1]. Education, culture, language, and the characteristics of the health-related setting all mediate one's capacity to process health related information [1]. In this paper we measured one component of health literacy, namely reading ability.

There is increasing evidence supporting an association between limited reading ability, and increased utilization of health care services [2,3], decreased use of preventive health care services [4-7], and poorer health outcomes in adults with chronic disease. [8-10]. These findings suggest an association between reading ability and the quality and outcomes of health care and provide an impetus to identify individuals with limited reading ability for targeted interventions. It is not known if limited reading ability is a marker for other factors that lead to poor health or if limited reading ability itself is a significant variable that directly affects health outcomes. The association between compensation for limited reading ability and improvement in health outcomes is also not known. However, tools to easily identify people with limited reading ability will help move this research agenda forward.

While recent studies describe a high prevalence of limited reading ability, literacy issues are difficult to identify during routine clinical care [11]. Successful screening for limited reading ability may help to identify people who need special methods of communication in clinical settings. Furthermore, it could increase the feasibility of clinical studies of literacy.

Current measures of health literacy primarily assess print literacy within health contexts. They are indicators of reading skills, and do not assess the full set of skills and knowledge associated with health literacy [1]. In addition, these instruments generally exclude patients who do not read the dominant local language or have low vision or other physical limitations that affect reading. Several instruments that measure reading ability are used in research studies, but the time required to administer these tools (from 3 minutes on average for the REALM-R [12] to 12 minutes for the TOFHLA. [13]) limits their usefulness in the practice environment. Two recent papers address screening for low literacy among adult caregivers of pediatric patients. Sanders and colleagues report that asking parents about the number of children's books in the home is a nonintrusive way of screening for adequate health literacy and has a positive predictive value (PPV) of 0.91 but it has a low negative predictive value (NPV) of 0.24. [14]. Bennett, Robbins and Haecker propose a set of 3 screening questions to identify risk of low literacy among adult caregivers of children. Two positive responses to these screening questions yields a sensitivity of 0.84 and a specificity of 0.56 using REALM score as the gold standard. [15]. These screening questions are limited to households with young children and need additional testing before widespread adoption.

Chew and colleagues. [16] evaluated 16 single screening questions to identify inadequate literacy in a preoperative Veterans Administration population. Three of the questions were effective at identifying individuals with inadequate reading ability as measured by the Short Test of Functional Health Literacy in Adults (S-TOFHLA) [13,17], a standard health literacy instrument. We modified these questions to develop a single item literacy screener (SILS) that would efficiently identify patients who have difficulty with a central aspect of health literacy, reading health related materials. The goal of this instrument is to identify patients who need help with written or printed material, regardless of the etiology (limited education, language barrier, physical impairment, *etc*.). We tested the performance of the SILS in a population more diverse than the Veterans Administration population used by Chew and colleagues. The aim of this study was to assess the diagnostic accuracy of the SILS as an indicator of limited reading ability, specifically the need for help reading or understanding printed health information, compared with a reference diagnostic strategy employing the Short Test of Functional Health Literacy in Adults (S-TOFHLA).

## Methods

This study was part of a larger project, the Vermont Diabetes Information System (VDIS), a cluster-randomized trial of a diabetes decision support system in a region-wide sample of Primary Care practices including 7406 patients with diabetes. [18]. A field survey targeted at a sub-sample of subjects aged 18 years or older was designed to provide a better understanding of the non-laboratory features of diabetes. Subjects were selected at random from the patients in each practice participating in the VDIS trial. They were invited by phone to participate in an in-home interview that included completion of a questionnaire and administration of the S-TOFHLA. Twelve research assistants, blinded to the reading ability of the subjects, were trained in the administration of the S-TOFHLA. Demographic information including age, sex, race, ethnicity, education, income, and marital status were also obtained. We attempted to contact 4,209 patients and reached 1,576. Of these, 64% agreed to be interviewed. Because of incomplete data on 8 subjects, 999 subjects were included in the analysis. The University of Vermont Institutional Review Board approved the study and all subjects gave written informed consent to participate in the interview.

The SILS is a single item question intended to identify adults in need of help with printed health material. The SILS asks, "How often do you need to have someone help you when you read instructions, pamphlets, or other written material from your doctor or pharmacy?" Possible responses are 1-Never, 2-Rarely, 3-Sometimes, 4-Often, and 5-Always. Scores greater than 2 were considered positive, indicating some difficulty with reading printed health related material. The cutoff off above 2 was chosen because we wanted to capture all who indicated they typically need help with written material and the sensitivity and specificity in this study were acceptable given the clinical trade-offs. In this study, the SILS was administered in written format as part of a questionnaire. Twenty-six patients with visual or other impairment had the SILS read to them. The SILS was always done prior to the S-TOFHLA.

The S-TOFHLA is a 36-item, 7-minute timed test of reading comprehension that we used as the reference measure of reading ability [17]. It employs the Cloze procedure in which a word in a sentence is omitted and must be chosen from a multiple choice list. The reading texts are passages from instructions for preparation for an upper gastrointestinal series and the patient "Rights and Responsibilities" section of a Medicaid application. Results are categorized into inadequate, marginal, or adequate health literacy (reading ability). Inadequate reading ability refers to a score of zero to 16 and represents individuals who often misread basic materials such as an appointment slip. Individuals scoring 17 to 22 have marginal reading ability and often have difficulty comprehending more complicated information such as that found in health educational pamphlets. Those with adequate reading ability score between 23 and 36 and typically are able to understand most printed health material [17]. The S-TOFHLA has demonstrated good internal consistency (Cronbach's alpha = 0.98 for all items combined) and concurrent validity compared to the long version of the TOFHLA (r = 0.91) and a medical-word recognition and pronunciation test, the Rapid Estimate of Adult Literacy in Medicine (REALM) (r = 0.80) [17,19].

Our goal was to develop a screening assessment that would cast a broad net to capture all subjects with limited reading ability. We therefore combined subjects who scored in the inadequate and marginal ranges on the S-TOFHLA (0 to 22 correct answers) or were unable to take the test because of visual or other impairments, into one group, which we refer to as "limited reading ability." All other subjects had "adequate reading ability" (23 to 36 correct answers on the S-TOFHLA). Visual impairment was defined by self-report. Our rationale for including subjects with visual or other impairments in our study was that subjects such as these would be among those who would be asked this screening question in a clinical setting. They are important to identify, as they are likely to require special methods of communication. Defining limited reading ability in this broader sense captures more potential challenges to communication in the clinical setting regardless of the etiology.

We used descriptive statistics, percentages, means and ranges, to describe the characteristics of the population. The diagnostic accuracy of SILS (the index test) compared to a S-TOFHLA score < 23 (the reference or "gold standard" test) was analyzed by calculating sensitivity, specificity, and predictive values [with their 95% confidence intervals (CI)], likelihood ratios (LR), as well as a receiver-operating characteristic (ROC) curve [20]. The sensitivity and specificity of an index test change as the decision threshold of the test is varied. The ROC curve represents these changes. We summarized the ROC curve by calculating the area under the curve (c-statistic), which ranges from 0.5 for an index test with no discriminatory ability to 1.0 for a test that is perfectly accurate. We did not focus on causation of limited reading ability and thus did not adjust for any confounders, limiting our analyses to understanding the screening value of the SILS. Data analyses were performed using STATA 8.2 (Stata Corporation, College Station, Texas).

## Results

The demographic characteristics of the study population were similar to the population of Vermont (Table 1). [21]. Of the 999 subjects screened, 170 (17%) had limited reading ability (S-TOFHLA score 0–22, blind, or otherwise unable to read) and 23% reported that they sometimes, often, or always need help with written health information (SILS>2).

**Table 1:** Subject characteristics

*Characteristic*	*N*	*Result*
Mean age in years, (range)	999	64.7 (22–93)
Female (N, %)	543	54
Race (N, % white)	969	97
Married [or living as married N, %]	625	63
Education (N, %)		
Less than High School	243	24
High School	353	35
Some College	159	16
Associate's Degree	59	6
Bachelor's Degree	87	9
Graduate or Professional Degree	91	9
Income (N, %)		
Less than $30,000 per year	542	59
$30,000 – $59,000 per year	259	28
$60,000 per year or more	123	13
Health Insurance^† ^(N, %)		
Private or commercial	581	58
Medicare	591	59
Medicaid	211	21
Military or Veterans Administration	51	5
None	24	2
Glycemic Monitoring and Control		
A1C (%), baseline mean (range)	992	7.1 (4–13.5)
Baseline A1C < 7% (N, %)	565	57
Reading Skill		
SILS (N, mean, range)	992	1.8 (1–5)
Never/Rarely needs help (N, %)	770	77
Sometimes need help (N, %)	147	15
Often need help (N, %)	39	4
Always need help (N, %)	43	4
S-TOFHLA^‡^, (N, mean, range)	999	29.8 (0–36)
Inadequate (%)	104	10
Marginal (%)	66	7
Adequate (%)	829	83

Some totals may not be 100% because of rounding.

^†^Many subjects had more than one health insurance type.

^‡^Inadequate (STOFHLA score 0–16, including the blind and those otherwise unable to read), Marginal (STOFHLA score 17–23), Adequate (STOFHLA score ≥ 23).

**Figure 1:**
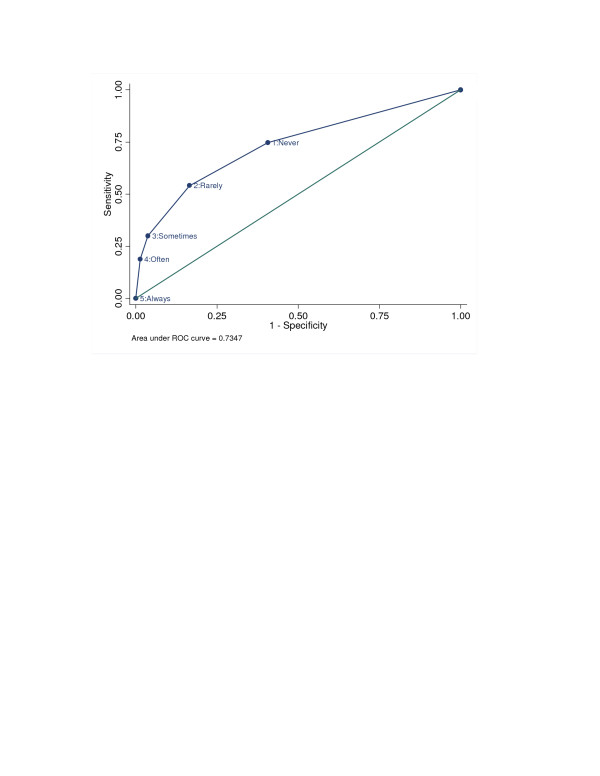
The Receiver Operating Characteristic Curve for SILS in detecting limited reading ability.

Comparisons between the sensitivity and specificity of the SILS using different thresholds to indicate a positive screen demonstrate the value of using a score greater than 2 as a positive finding. (Table 2). Lowering the threshold increases the sensitivity but decreases the specificity. A threshold of SILS>2 appears to provide the best trade-off with a sensitivity of 54% and a specificity of 83%. The area under the Receiver Operating Characteristics Curve (Figure 1) was 0.73 (95% confidence interval 0.69, 0.78). Using a score > 2 on the SILS, the LR+ is 3.2 and the LR- is 0.6. This positive likelihood ratio suggests a moderate ability to discriminate between patients with and without limited reading ability.

**Table 2:** 2

*SILS Threshold*	*Sensitivity, % (95% CI)*	*Specificity, % (95% CI)*	*Likelihood Ratio Positive*	*Likelihood Ratio Negative*	*Yield %*
>1	75 (68, 81)	59 (56, 63)	1.83 (1.63, 2.07)	0.42 (0.33, 0.56)	46
>2	54 (47, 61)	83 (81, 86)	3.18 (2.67, 4.03)	0.55 (0.47, 0.65)	23
>3	30 (24, 37)	96 (95, 97)	8.02 (5.30, 12.15)	0.73 (0.66, 0.80)	8

Performance of SILS at different thresholds (N = 999)Reference test is STOFHLA, score < 23 is positive for limited reading ability. Results are point estimates and 95% confidence intervals. Yield reflects the percentage of the population at or above the threshold.

Of the 229 subjects who scored positive on the SILS (SILS > 2), 40% had limited reading ability (S-TOFHLA score < 23 or otherwise impaired) (Table 3). Seventy-eight percent screened negative on the SILS (score ≤ 2). Of these, 90% had adequate reading ability as measured by the S-TOFHLA.

**Table 3:** Distribution of subjects on the reference test (S-TOFHLA) and the SILS

	*Limited Reading Ability*			*Adequate*	*Total*
	Impaired	*STOFHLA 0–16*	*STOFHLA 17–22*	*STOFHLA 23–26*	
SILS>2 (positive)	23	47	22	137	229
SILS <2 (negative)	3	31	44	692	770
*Total*	26	78	66	829	999

Impaired = Low vision or physically unable to complete the S-TOFHLA.

The SILS had better performance in the subset of patients with the lowest reading ability (Table 4). A SILS score greater than 2 had a 67% sensitivity (82% specificity) for subjects with S-TOFHLA scores less than 16 or with low vision or physically unable to complete the S-TOFHLA. The majority of subjects with limited reading ability not detected by the SILS (false negative subjects) had milder deficits on the reference test (S-TOFHLA > 16). Forty-four of the 78 false-negative patients (56%) had marginal rather than inadequate reading ability. A SILS score greater than 2 detected 33% of the subjects with marginal reading ability as measured by the S-TOFHLA (score between 17–22).

**Table 4:** Performance of SILS at a threshold of 2 (N = 999)*

***Characteristic***	*Impaired*^†^*and Inadequate Reading Ability (S-TOFHLA < 16)*^‡^	*Impaired*^†^*and Limited Reading Ability (S-TOFHLA < 23)*^‡^
Sensitivity (%)	67 (58, 76)	54 (47, 61)
Specificity (%)	82 (80, 85)	83 (81, 86)
Positive Predictive Power	0.31 (0.25, 0.37)	0.40 (0.34, 0.47)
Negative Predictive Power	0.96 (0.94, 0.97)	0.90 (0.88, 0.92)
Likelihood Ratio Positive (LR +)	3.79 (3.12, 4.6)	3.28 (2.67, 4.03)
Likelihood Ratio Negative (LR -)	0.40 (0.30, 0.53)	0.55 (0.47, 0.65)
Area under the ROC curve	0.78 (0.73,0.83)	0.73 (0.69, 0.78)

**Main analysis is based on S-TOFHLA < 23.

^†^Impaired = Low vision or physically unable to complete the S-TOFHLA.

^‡^Results are point estimates and 95% confidence intervals.

## Discussion

The SILS is a single item instrument for the identification of patients who need help with reading health related information. In this population, the SILS performs reasonably well. The S-TOFHLA takes up to seven minutes but the SILS is very brief and therefore practical for use during a routine clinical encounter.

Our finding of 17% prevalence of limited reading ability in an older population with chronic disease is lower than a recent pooled analyses of prevalence studies [22] which reveal a weighted prevalence of low reading ability of 26% (95% confidence interval [CI] 22–29%) and of marginal reading ability of 20% (95% CI, 16%–23%). In the pooled analysis, level of education, ethnicity, and age were all associated with low reading ability. Our more educated and ethnically homogenous population may explain some of this difference.

Similar to Chew and colleagues'. [16] single item questions, the SILS did not perform as well (sensitivity of 34%) for patients with marginal reading ability (S-TOFHLA scores 17–22). These false negative results may be because subjects may not recognize that they need help with reading, may be ashamed of a literacy problem. [23], or may simply not understand the question.

The SILS had a larger area under the ROC curve for limited reading ability (the combination of impaired, inadequate or marginal reading ability) than any of the three questions proposed by Chew *et al*. [16] (c = 0.73 *vs*. 0.68, 0.66, and 0.60). Although the area under the ROC curve is higher if we do not include those with marginal reading ability, we propose that this group of patients is also in need of additional assessment of their reading ability and potentially alternative methods of communication to optimize care.

Our sensitivity and specificity are similar to those reported by Bennett et al [15] for the three item screening questions that they evaluated for use with adult caregivers of pediatric patients. These results add support to the feasibility of screening for reading difficulties in the clinical practice setting.

We chose the S-TOFHLA as the gold standard for this analysis because it is among the most widely used instruments for the assessment of health literacy. We would not expect, though, a perfect correlation between the two instruments. The SILS is measuring something distinct from reading ability, that is, the need for help with reading health-related materials. It is quite plausible, for example, that a person with adequate general literacy (and an adequate score on the S-TOFHLA), would routinely "need help" reading complex health information. If the intent is to determine who actually needs help reading health-related materials, the SILS is a more direct measure than the STOFHLA. More research is needed to understand the differences between populations identified by the SILS and the S-TOFHLA.

We envision the SILS being asked routinely at the time of patient registration or with the vital signs, as a potential first step to engage a patient in a dialogue about improving health related communication. Education and ethnicity have been reported to be significantly associated with health literacy [6,8-10,24,25] and some may argue that these factors could be used to identify patients who are most in need of alternative communication strategies, rather than a new instrument. However, the SILS is a more direct assessment of a need and is simpler than an estimate based on demographic or cultural factors. Asking directly may also identify those with limited reading ability who already have a satisfactory compensatory strategy in place eliminating the need for further assessment.

This study has several limitations. The subjects were recipients of health care for diabetes in a single region of the United States, and may not be representative of patients from other areas. Most subjects had health insurance which reflects the fact that they were recruited from medical practices. A few subjects had the SILS read aloud to them while the majority responded to the SILS on paper, which may decrease the accuracy of the SILS in detecting limited reading ability. Future studies are needed to compare the performance of the SILS when read aloud versus administered on paper. Although our study sample is limited to a single region and is racially homogenous, all the subjects were outpatients with a chronic illness and should be representative of patients cared for in many community primary care settings in the U.S. We chose to use a cutoff of > 2 as in indicator of a positive SILS for this study, however in settings where the goal is to maximize sensitivity, using a threshold of > 1 should be strongly considered.

## Conclusion

In this primary care population, one in six had limited reading ability. With the known negative impact of limited reading ability on health outcomes, enhancing communication for this population is critical. The SILS performs moderately well at ruling out limited reading ability in adults and allows providers to target additional assessment to those most in need. Application of the SILS in clinical settings has the potential to improve outcomes and processes of care for chronically ill individuals with limited reading ability.

## Competing interests

The author(s) declare that they have no competing interests.

## Authors' contributions

NSM made substantial contributions to analysis and interpretation of data, provided important intellectual content, and was involved in drafting and revising the manuscript. CDM made substantial contributions to conception and design; acquisition, analysis and interpretation of data; provided intellectual content and contributed to critical revision of manuscript. LC contributed to critical revisions and provided important intellectual content. BL made substantial contributions to conception and design; acquisition, analysis and interpretation of data; provided intellectual content and contributed to critical revision of manuscript.

## Pre-publication history

The pre-publication history for this paper can be accessed here:


